# Dilatation of the Stomach

**Published:** 1889-03

**Authors:** J. Michell Clarke

**Affiliations:** Assistant-Physician, Bristol General Hospital; and Assistant-Lecturer on Physiology, Bristol Medical School


### DILATATION OF THE STOMACH.

BY

J. Michell Clarke, M.A., M.B. Cantab., M.R.C.P.

*Assistant-Physician, Bristol General Hospital; and Assistant-Lecturer on Physiology, Bristol Medical School.*

---

Dilatation of the stomach is not uncommon, and gives rise to a very troublesome form of dyspepsia. I propose in this paper to study the causes of dilatation, and the measures best adapted for its relief. The condition may be produced either by an obstruction at the pylorus to the expulsion of the gastric contents, or by morbid changes in the muscular walls of the stomach without pyloric obstruction. Stenosis of the pylorus is most commonly due to cancer, but may also be produced by simple fibrous stricture, or by the cicatricial contraction resulting from the presence of an ulcer in this situation. I do not propose to deal with this group of cases, in which the conditions present do not, unfortunately, admit of cure.

As to the etiology of other varieties of dilatation, two chief theories have been advanced: (1) that the stomach is the primary cause of the disease, other symptoms being secondary; and (2) that there is a general neurasthenic tendency, in which the stomach suffers in common with other organs.

The actual mechanism of gastric dilatation has been attributed to the following causes: (1) to hereditary weakness of the gastric muscle;* (2) to gastritis,

* Bouchard, *Leçons sur les Auto-intoxications.*

which may go on to sclerosis of the gastric walls: under this head may be included the abuse of alcohol, which sets up degenerative changes resulting in atrophy of glandular structures and fatty degeneration of muscle. Gastric ectasia sometimes occurs after long continued high fever and in severe anæmia; and it is suggested that, just as anæmia gives rise to fatty degeneration of heart-muscle, followed by dilatation of the cardiac cavities, it may in a similar fashion produce dilatation of the stomach. It is important to bear in mind that this condition may occur in the later stages of typhoid fever, and protract convalescence.

One case that recently came under my observation seems to show that it may occasionally lead to death. A patient died unexpectedly in the fifth week of the disease, apparently from syncope; the fever had run a mild course, and she was progressing favourably towards convalescence. At the autopsy, the only cause to which death could be attributed was that the stomach was enormously distended with gas, and had pushed up the diaphragm and, presumably, thus interfered with the action of the heart.

(3) Glénard of Lyons states that in women suffering from dyspepsia after childbirth, and sometimes in other persons, a condition which he calls "enteroptosis" obtains; that is to say, the mesenteric attachments of the transverse colon are relaxed, it tends to fall forwards, and drags with it the stomach, which undergoes atony and dilatation, and may even be actually lowered in position.

(4) In nearly fifty per cent. of cases of gastric ectasia, the gastric juice contains an excess of hydrochloric acid. Kussmaul and others think that this hyperacidity may lead to spasm of the pylorus, and that this spasm, constantly recurring and opposing the passage of food through the pylorus, produces dilatation. Lastly, antecedent dyspepsia, bringing about too long sojourn of the food in the stomach; irregular or too frequent meals; and the ingestion of large quantities of heavy food with much indigestible residue, or of food insufficiently masticated, may all lead to dilatation.

In support of the second theory above mentioned, that gastric ectasia is merely a part of a general neuropathic tendency, in which the stomach shares—the ancient hypochondria,— it may be urged that the symptoms of neurasthenia, and of certain forms of gastric dyspepsia, are very similar, that both conditions frequently co-exist, and that it is often exceedingly difficult to assign them to their respective causes.

Judging from my own experience, I should say that in some cases the stomach appears to be alone affected. The condition is sometimes found in fairly strong men of the working class, who are accustomed to eat large meals of heavy food with much indigestible residue; the stomach, in fact, gradually gives way under the weight of the mass of food that it has to support. In other patients the condition of general weakness is equally striking: the muscles are everywhere weak, soft, and flabby; their motor force is impaired, whilst their irritability is in marked excess, as shown by the readiness with which they respond to a tap upon their mass or on their tendons, whilst the pectorals often show the phenomenon of "myoidema." In these cases we may suppose that the gastric muscle shares in the general muscular weakness, and so allows dilatation to occur.

For examination, the patient should lie on the back, with the knees drawn up, breathing quietly with opened mouth and relaxed abdominal parietes. If there is gastric dilatation, a distension of the upper part of the abdomen, or peristaltic movements of the organ, may be observed. The area of stomach-resonance is enlarged: this normally should not extend higher than the sixth rib; its average vertical diameter in the adult male is 5 inches; in the female, 4½ inches; and the transverse diameter is 8½ inches (male), 7½ (female).* A splashing sound may be heard on succussion of the patient. More importance is to be attached to what has been termed "partial succussion." Placing the hands upon the abdomen, a splashing sound is evoked by sharply depressing the fingers of either hand alternately. If this splash is obtained at any time below a line drawn from the umbilicus to the salient angle of the false ribs on the left side, or if it is obtained anywhere over the stomach when from five to six hours have elapsed since the last meal, there is a pathological degree of enlargement of the stomach.

During fasting, when an increased area of resonance, but no splash, is fonnd on examination, information may be gained by letting the patient drink a glass or two of water: a zone dull on percussion occupying the lower part of the previously tympanitic region at or below the level of the umbilicus (on the left side), indicates dilatation. Very often there is great tenderness over a spot just below and to the right of the ensiform cartilage. Other procedures, such as the injection of effervescing mixtures, or the passage of a bougie, are objectionable, from the discomfort they cause the patient. The administration of salol, gr. xv., is a useful aid in determining the length of time that food stays in the stomach: this substance, a

* Mathien, *Gazette des hôpitaux*, Feb., 1888.

compound of salicylic acid and phenol, is decomposed by the pancreatic, but not by the gastric juice. One hour after its ingestion, salicylic acid should normally be detected in the urine by the perchloride of iron reaction : in cases of dilatation, this reaction may not be obtained until one and a half, or even two or three hours after ingestion.*

These procedures enable us to state that the stomach is dilated, and that fluids stay in it too long a time; they may be supplemented by chemical examination of the gastric contents, withdrawn by a syphon.

Dilatation of the stomach may come on at any age, but is most common during adult life; of twenty-two cases lately under my care, eleven of the patients were between forty and sixty years of age. Several of these patients were accustomed to eat large meals of coarse food; one dated the onset of the illness from an attack of enteric fever, and another from blood-poisoning after eating some tainted beef. The symptoms fall into two sets—"direct," due to disturbance of the alimentary canal, and "remote." Of the former, the patients complain of pain and sensation of weight at the epigastrium, coming on at a varying time after meals, or constantly present day and night, and aggravated by food. They often suffer from pyrosis, and from bitter or fœtid eructations two or three hours after meals. Flatulence is present in most cases, and may be extreme. The pain may be felt chiefly in the back and sides. In the majority of cases there is no sickness, and the appetite may be good, or even voracious. There is generally constipation, sometimes alternated with the passage of loose fœtid evacuations. Attacks of colicky pain coming on during the night, and waking the patient, are characteristic: they are generally

* Mathien, *loc. cit.*

accompanied with excessive distension of the stomach, and sometimes with shivering, pallor of the skin, coldness of extremities and violent palpitation.

Gastric ectasia is often associated with pulmonary phthisis, chlorosis, and hypochondriasis: Bouchard* thinks that it predisposes to these affections by interfering with nutrition.

As to the remote symptoms: drowsiness, headache, depression of spirits, vertigo, feebleness, palpitation, and all the numerous symptoms of this kind are frequent. Asthmatic attacks are not uncommon. Albumen is present in the urine—rarely exceeding a trace—in about 15 per cent. of the cases; it promptly disappears under treatment. Sometimes there is peptonuria. Bouchard lays stress on the occasional presence of a body, as in some cases of diabetes, which gives a chloroform-like odour to the breath, and a vinous red colour on the addition of perchloride of iron to the urine. He also draws attention to the occurrence of nodosities resembling Heberden's, but situated at the bases of the second phalanges of the fingers instead of the first: I have observed these nodosities in four cases. A species of fainting fit may occur, attributable, perhaps, to a neurosis of the vagus. For instance, a man of 29, whilst reading, felt as if his chest were suddenly and forcibly gripped; he believed that he lost consciousness for a few moments. The attack was preceded and followed by palpitation, and also followed by flatulent distension of the abdomen. A few days later, another similar attack occurred, attended with a stabbing pain in the head, but without loss of consciousness. Under treatment for the stomach-condition, these symptoms at once ceased, and did not recur.

* *Loc. cit.*

Lastly, gastralgia coming on at the commencement of gastric digestion, may give rise to such violent pain as to simulate biliary colic.

In diagnosis, it is often difficult, and sometimes impossible, to determine the exact condition present. The presence of a tumour, an enormous degree of dilatation which progressively increases, and the absence of hydrochloric acid from the gastric juice, point to organic disease. In atonic functional ectasia, we generally find a lesser degree of dilatation, and this varying in extent from time to time, with a normal or excessive quantity of hydrochloric acid in the gastric juice. M. Mathien considers that the reduction of the stomach-area of resonance under the influence of ipecacuanha, is the most important element in prognosis. Should the degree of dilatation remain unchanged after treatment has been persevered in for some time, the inference may be drawn that the muscular tissue is degenerated.*

Although failure of the nervo-motor functions of the stomach is probably the primary cause of dilatation, it is soon attended with disorder of the chemical processes of digestion. The presence of food in the stomach later than five hours after ingestion, is pathological; in about six hours fermentative changes begin, and after seven hours the alimentary residue will undergo exclusively acid or putrid fermentations.† The products of these fermentative changes will tend to still further irritate and distend the stomach, and being absorbed, to give rise to auto-intoxication.

The indications for treatment are, therefore: (1) to evacuate the contents of the stomach, if it is no longer able to propel them into the duodenum; (2) to give an

* *Le Progrès médical.* February, 1887. † Bouchard, *loc. cit.*

appropriate diet; (3) to check anomalous fermentations, and so prevent auto-intoxication; (4) to excite the contractility of the gastric muscle; and (5) to regulate the action of the bowels and improve the general health.

In the class of cases now under consideration, in which the dilatation is more or less curable, and not due to organic disease, evacuation of the stomach-contents and lavage are not, as a rule, necessary. The procedure is merely palliative; it has the disadvantages of diminishing appetite and digestion, and is disagreeable to most patients. In the severer cases, however, it gives relief at the commencement of treatment, and may be afterwards discontinued; and in acute dilatation, *e.g.* from the ingestion of putrid meat, or of other injurious food, the stomach is to be at once evacuated.

In regulating the diet, the object in view is to distend the stomach as rarely, as little, and for as short a time as possible. There must be no eating or drinking between meals. The food must be finely divided, capable of thorough mastication, and must leave little indigestible residue. Bouchard, following the plan formerly advocated by Chomel, reduces the quantity of fluid taken to a minimum, in order not to dilute the gastric juice. Meat, eggs, fish, and milk, with crust or toast, are the best articles of diet; rice, barley, and oatmeal, are generally well digested: no green vegetables are allowed, and fats, except in the form of emulsion, are best avoided. Alcohol and the soft parts of bread are especially liable to give rise to fermentative changes. Wine, if taken, is to be diluted with water; a small quantity of light beer, or a dessert-spoonful of brandy or whisky, may be allowed; tea and coffee, unless very weak, are best avoided.

Dujardin-Beaumetz * divides the patients into two categories—those with constipation, and those with diarrhœa. For the latter he forbids meat and eggs, and orders a purely vegetable diet of starchy foods—vegetables and fruits, care being taken that the vegetables are tender, finely divided, and well cooked.

The meals are to be few and far between—substantial but not copious; the patient should rest a short time after each meal. They may be three in number: the first between 8 and 9 a.m., the second at 1 p.m., and the third at 7.30 p.m. In treating a case of ordinary severity, I generally recommend at first the following diet: for breakfast, milk, bread and milk, porridge, or an egg in milk. For dinner, either fish, milk pudding, with toast or crust of bread, or bread and milk. For supper the same, varying the articles of food. If, after a few days, the patient is free from pain, the dietary is cautiously extended by the gradual addition of chicken, game, or meat, until a diet something like the following is finally adopted:—for the first meal, egg or fish and dry toast, with a small teacupful of weak coffee or cocoa and milk: for the second and third meals, boiled fish, poultry, or well-cooked hot or cold meats, eggs, custard or milk puddings, with a small quantity of vegetables and bread; cooked fruit may be taken; of fresh fruit, strawberries, peaches, grapes, oranges (the juice), are alone advisable. 6—8 oz. of fluid is allowed at each of the two last meals. To make up for the deficiency of fluids, and as a stimulant to the intestinal movements, the patients are recommended to take a full glass of hot water at night, to which ʒij. of whisky or brandy may be added, if desired. In the more severe cases, it is better to keep the patient entirely on milk for

* *Therapeutic Gazette.* January, 1887.

a few days; if neither milk nor farinaceous food can be digested, peptonised milk is to be employed, and given in small quantity every hour. As improvement takes place, the quantity taken at a time is increased, and given less often, and the peptonised is gradually replaced by ordinary milk. Then the yolk of an egg may be taken, beaten up in milk, two or three times a day; raw oysters are, as a rule, well digested. Later, preparations of rice, barley, sago, or oatmeal, take the place of some of the milk, and the dietary scale is now steadily extended. In these patients rest in bed for from seven to fourteen days is most important, and materially shortens the duration of treatment.

For the relief of pain, hot drinks, chloroform water, cocaine, a liquid preparation of bismuth with dilute hydrocyanic acid or liquor morphiæ, ♏v. in hot water, are useful. Sometimes the solid subnitrate of bismuth answers best, and arsenic is often valuable. If the pain is not great, a mixture containing dilute hydrochloric acid, with strychnine and one of the liquid preparations of pepsine in chloroform-water, taken after meals, aids digestion and checks fermentation. For the latter purpose, Dujardin-Beaumetz* employs carbon disulphide water: $\beta$ naphthol and sulpho-carbolate of soda may also be used. Acids must not be given if there is any suspicion of gastric ulcer. Of the remedies employed with the object of exciting the contractility of the gastric muscle, ipecacuanha, originally recommended by Mathien, takes the first place. It may be given in doses of gr. $\frac{1}{2}$ to gr. ij., in powder, half an hour after meals, two or three times a day. I generally give gr. j., in milk, half an hour before rising and at bedtime, increasing the dose to gr. ij. if it is well borne. Strychnine

* For mode of preparation see *Therapeutic Gazette*, January 15, 1887.

is also of service, and may be advantageously combined with bismuth, and given in hot water, since hot fluids stimulate the gastric muscle and relieve pain. Electricity is useless.

As to laxatives, rhubarb, compound liquorice powder, cascara sagrada, podophyllin and nux vomica, or the use of small enemata with a dose of ʒij.—ʒiij. of glycerine, taken in water before breakfast, are amongst the most useful. Cold baths and change of air are the best general tonics.

In conclusion, patients who faithfully carry out the above rules, generally get relief from pain in two or three days, even after years of illness: the amelioration is complete during treatment, and they are often apparently cured in three or four weeks; but it is necessary to warn them that a relapse will occur unless they keep to the prescribed diet, to which they should adhere for from one to two years.

